# Current topics and management of head and neck sarcomas

**DOI:** 10.1093/jjco/hyad048

**Published:** 2023-06-10

**Authors:** Kenya Kobayashi, Nobuhiro Hanai, Seiichi Yoshimoto, Yuki Saito, Akihiro Homma

**Affiliations:** Department of Otolaryngology–Head and Neck Surgery, University of Tokyo, Tokyo; Department of Head and Neck Surgery, Aichi Cancer Center Hospital, Nagoya; Department of Head and Neck Surgery, National Cancer Center Hospital, Tokyo; Department of Otolaryngology–Head and Neck Surgery, University of Tokyo, Tokyo; Department of Otolaryngology–Head and Neck Surgery, Faculty of Medicine and Graduate School of Medicine, Hokkaido University, Sapporo, Japan

**Keywords:** rhabdomyosarcoma, Ewing’s sarcoma, osteosarcoma, angiosarcoma, chemotherapy, radiotherapy

## Abstract

Given the low incidence, variety of histological types, and heterogeneous biological features of head and neck sarcomas, there is limited high-quality evidence available to head and neck oncologists. For resectable sarcomas, surgical resection followed by radiotherapy is the principle of local treatment, and perioperative chemotherapy is considered for chemotherapy-sensitive sarcomas. They often originate in anatomical border areas such as the skull base and mediastinum, and they require a multidisciplinary treatment approach considering functional and cosmetic impairment. Moreover, head and neck sarcomas may exhibit different behaviour and characteristics than sarcomas of other areas. In recent years, the molecular biological features of sarcomas have been used for the pathological diagnosis and development of novel agents. This review describes the historical background and recent topics that head and neck oncologists should know about this rare tumour from the following five perspectives: (i) epidemiology and general characteristics of head and neck sarcomas; (ii) changes in histopathological diagnosis in the genomic era; (iii) current standard treatment by histological type and clinical questions specific to head and neck; (iv) new drugs for advanced and metastatic soft tissue sarcomas; and (v) proton and carbon ion radiotherapy for head and neck sarcomas.

## Teaser

Head and neck sarcomas are rare, histologically diverse tumours. This review describes the historical background and recent topics that head and neck oncologists should know about this rare tumour.

## Introduction

Sarcomas are malignant solid tumours arising in the mesenchymal tissue and account for <1% of all malignant tumours. Of these, only 5–15% occur in the head and neck region, making it an extremely rare tumour (1,2). Owing to the low incidence and diversity of histological types of head and neck sarcomas, conducting large prospective studies is challenging; therefore, it is difficult to obtain high-quality evidence (3). Treatment strategies are often drawn from the evidence obtained in other anatomical regions or paediatrics. A multidisciplinary team is essential for treatment, including head and neck surgeons, medical oncologists, paediatricians, radiologists, radiation oncologists and pathologists (3).

To comprehend the principles of surgical treatment for head and neck sarcomas, head and neck oncologists should understand the concept of multidisciplinary treatment of sarcomas. For resectable sarcomas, surgical resection followed by radiotherapy (RT) is considered the standard of care. Tumours often originate in anatomical borderline regions such as the skull base or mediastinum and require reconstruction after resection; therefore, it is often necessary to consult with plastic reconstructive surgeons, neurosurgeons, thoracic surgeons and orthopaedic surgeons. In head and neck sarcomas, securing an adequate margin is challenging. Extensive resection decreases the quality of life of the patients, and perioperative chemotherapy is indicated for round cell sarcomas [such as rhabdomyosarcomas (RMS) and Ewing’s sarcoma] and osteosarcomas, which are sensitive to chemotherapy.

In advanced or metastatic sarcomas, established regimens for each histological type are adapted for chemo-sensitive round cell sarcomas (RMS and Ewing’s sarcoma) and osteosarcomas. Although doxorubicin alone has been the standard regimen for advanced or metastatic soft tissue sarcomas with no established standard chemotherapy (4), in recent years, the efficacy of novel agents such as pazopanib, trabectedin and eribulin has been established in non-round cell sarcomas such as liposarcomas, leiomyosarcomas and synovial sarcomas, which are less sensitive to chemotherapy (5–7).

In this review, we will discuss the historical background and recent topics in the development of the treatment of head and neck sarcomas from the following five perspectives: (i) epidemiology and general characteristics of head and neck sarcomas; (ii) changes in the histopathological diagnosis in the genomic era; (iii) current standard of treatment by histological types and clinical questions specific to the head and neck; (iv) novel drugs for advanced or metastatic soft tissue sarcomas; and (v) proton and carbon ion RT for head and neck sarcomas.

## Epidemiology and general characteristics of head and neck sarcomas

Sarcomas arise in any soft tissue or osseous tissues of the region and may be found in any person, regardless of age or sex. Head and neck sarcomas represent a pathologically heterogeneous group of malignancies. As regards specific histological types of sarcomas, the relative incidence is significantly influenced by patient age and anatomical location (8–10).

The National Cancer Institute’s Surveillance of 12 725 head and neck sarcoma patients from 1973 to 2010 in the USA included 11 481 adults and 1244 paediatric patients (11). In the adult cohort, males predominated, with 8330 cases (73%), whereas females accounted for only 3151 cases (27%). The median age group affected was 55–59 years. The most common primary site was the skin and soft tissues (*n* = 7244, 63%), followed by the bones of the skull and face (*n* = 1277, 11%) and the oral cavity (*n* = 1108, 10%). The most common histological types were malignant fibrous histiocytomas (MFH), Kaposi sarcoma and hemangiosarcomas. In the paediatric cohort, males and females were similarly affected, with 659 cases (53%) reported in males and 585 cases (47%) in females. The median age group affected was 5–9 years. The most common primary site was the skin and soft tissues (*n* = 576, 46%), followed by the bones of the skull and face (*n* = 260, 21%) and the nasal cavities, paranasal sinuses and middle ear (*n* = 157, 12.6%). The most common histological types were RMS, MFH and osteosarcomas.

By primary locations, liposarcomas, malignant peripheral nerve sheath tumours and synovial sarcomas predominate in the neck; angiosarcomas and dermatofibrosarcomas in the scalp and facial skin; malignant peripheral nerve sheath tumours, myxofibrosarcomas and RMS in the nasal cavity and paranasal sinuses; and leiomyosarcomas and RMS in the oral cavity (12–14).

Unfortunately, head and neck sarcomas do not traditionally exhibit the high local control rates seen in other anatomical regions (3). This has been attributed to the traditional inability to deliver adequate intensive treatments because of their location among the critical structures in the head and neck region. Lymph node metastasis is relatively rare in most sarcomas (15), with an incidence of less than 14% (16–18). The rate of lymph node metastasis varies greatly by histological type. The sarcomas with the highest rate of lymph node metastasis were RMS (37–25%), clear cell sarcoma (38–16%), angiosarcoma(20–6%), epithelioid sarcoma (13–12%) and Ewing’s sarcoma of soft tissue (16%) (16–18). The benefits of prophylactic neck dissection in high-risk cases have not been fully established (18). Rarely, bone sarcomas may also present with regional lymph node disease, which carries a very unfavourable prognosis (19). Most sarcomas present with localized disease, with distant metastasis being present in <10% of cases at the time of diagnosis and initial treatment (3). Lung is the most frequent site of metastasis.

## Changes in histopathological diagnosis in the genomic era

**Figure 1:**
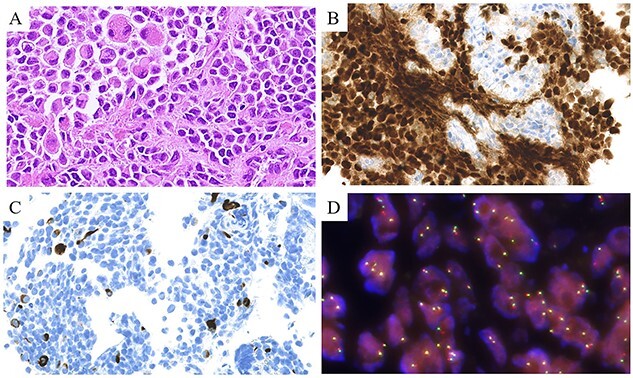
Pathological findings of alveolar rhabdomyosarcoma. (A) HE staining. Primitive, small, round cells appear to ‘float’ in a nest outlined by fibrous septa. (B) The nuclear expression of myogenin was 80%. (C) Desmin-positive cells are scattered. (D) The *PAX3-FOXO1* fusion FISH probe. The fusion gene is detected as a yellow signal, with the green and red signals confocalized.

Preoperative pathological diagnosis of sarcomas requires multifaceted analysis and tends to be time consuming; thus, the clinician should formulate a quick and efficient biopsy plan. The quality and quantity of biopsy specimens are critical in making a reliable pathological diagnosis. If necessary, an open biopsy should be performed without hesitation (20). Tumour cells can spread and implant along tissue planes facilitated by extravasated blood from the surgical site, and biopsy should be accomplished through the smallest incision possible with absolute haemostasis (3). To minimize the risk of biopsy seeding, the biopsy tract should be excised afterward or may be included in the RT target volume of postoperative treatment (3). Open biopsy is normally recommended because it provides sufficient tissue for traditional histological analysis, immunohistochemistry, molecular assessment and genomic analysis (Fig. 1). Given the recent technological innovations in molecular biological analysis, genetic aberrations in sarcomas have contributed to their diagnostic significance. Gene rearrangement and amplification can be detected by fluorescence *in situ* hybridization as a split or amplified signal, which is widely used in the routine pathological diagnosis of sarcomas. *EWSR1-FLI1* and *EWSR1-ERG* rearrangements in Ewing’s sarcoma (21,22), *DDIT3* gene rearrangements in myxoid liposarcomas (23), well-differentiated/dedifferentiated liposarcomas, *MDM2* amplification in parosteal osteosarcomas and low-grade central osteosarcomas (24), and *PAX3–FOXO1* and *PAX7–FOXO1* rearrangement in alveolar RMS (25) are extremely important for diagnosis and prognostic estimation. Although studies are limited to malignancies in the head and neck region, recently, core needle biopsy (CNB) has also been applied for histopathological diagnosis (26). CNB allows for assessing tissue architecture, grading, typing and analysing genomic status. As regards diagnostic value, CNB is similar to open biopsy (26). Sample quality is a factor that confounds histopathological diagnosis by CNB (27), and clinicians must reliably collect tumour tissue samples under ultrasound guidance. A successful pathological diagnosis by CNB depends on the abundant diagnostic experience of the pathologist and clinician (28). The actual incidence of tumour seeding along the cutting-needle tract in intraperitoneal, retroperitoneal and extremity sarcomas is <1% and usually has no prognostic relevance (29). The clinicians should also provide detailed clinical information to the pathologist. Age, primary site, progression speed and imaging findings strongly support an accurate pathological diagnosis. A close dialogue between the clinician and the pathologist is important in enhancing diagnostic accuracy (30,31).

## Current standard treatment by histological subtype and clinical questions specific to the head and neck

(1) RMS

RMS predominantly affects children and adolescents, and evidence of its treatment has been established mainly in children. Despite the relatively poor prognosis of adult RMS compared with paediatric RMS, adult patients whose treatment adhered to current guidelines for children had outcomes similar to those reported in the paediatric series, suggesting that adults and children with RMS should receive similar treatment (32). Historically, the treatment of RMS had been developed by two clinical trial groups: the Children Oncology Group’s Soft-Tissue Sarcoma Study Committee (COG-STS) in the USA and the European Pediatric Soft Tissue Sarcoma Study Group (EpSSG) in Europe.

In the USA, the Intergroup Rhabdomyosarcoma Study Group (IRSG), which is the parent organization of the COG-STS, conducted four consecutive trials (IRS-I, IRS-II, IRS-III and IRS-IV) (33–36). The survival rate was <25% prior to the consistent use of multi-agent chemotherapy beginning in the early 1970s (37). The overall survival (OS) rate for patients with localized RMS has improved from 55% on IRS-1 to 71% on IRS-IV (33,36). With the formation of the COG in 2000, the COG-STS conducted clinical trials (D9602, D9803 and D9802) for ‘low-risk’ (38), ‘intermediate-risk’ (39) and ‘high-risk’ (40) RMS. These studies were conducted as separate studies based on clinical and biological prognostic factors using risk stratification. Risk stratification for RMS is based on both a pretreatment (TNM) staging system and a surgical/pathologic clinical grouping system established by the IRSG/COG-STS (Table 1) (33,41,42). The clinical group is determined after the initial surgical procedure before the initiation of systemic therapy and is primarily based on the extent of the residual tumour and regional lymph node involvement after surgery. The COG-STS group system is useful in selecting treatment intensity and is highly predictive of outcomes (35). Thus, the treatment concept of COG-STS is a multidisciplinary approach based on risk stratification in the initial surgical resection. As a systemic chemotherapy, vincristine, actinomycin-D and cyclophosphamide (VAC) or vincristine and actinomycin-D (VA) is the established standard regimen. Despite randomized studies, systemic chemotherapy for most patients has largely remained unchanged since the 1970s. The appropriate dose of cyclophosphamide, which is associated with acute toxicity and reduced fertility, has historically been an unresolved issue, and clinical trials are ongoing to determine acceptable dose reductions based on risk stratification (43).

**Table 1:** Risk stratification for rhabdomyosarcoma

IRS Clinical Group Classification					
Group	Definition				
Group I	Localized disease, completely resected				
Group II	Total gross resection, with evidence of regional spread				
A	Grossly resected tumour with microscopic residual disease				
B	Involved regional nodes completely resected with no microscopic residual disease				
C	Involved regional nodes grossly resected with evidence of microscopic residual disease				
Group III	Biopsy only or incomplete resection with gross residual diseases				
Group IV	Distant metastatic disease (excludes regional nodes and adjacent organ infiltration)				
TNM Pretreatment Staging Classification					
Stage	Site	T^a^	Size^b^	Nodes^c^	Metastasis^d^
1	Orbit, head and neck (non-PM), GU (non-B/P), biliary tract	T1 or T2	a or b	N0 or N1 or Nx	M0
2	B/P, extremity, PM, other (includes trunk, retroperitoneum, etc.)	T1 or T2	a	N0 or Nx	M0
3	B/P, extremity, PM, other (includes trunk, retroperitoneum, etc.)	T1 or T2	a	N1	M0
			b	N0 or N1 or Nx	M0
4	Any	T1 or T2	a or b	N0 or N1 or Nx	M1

PM, parameningeal; B/P, bladder/prostate

a T: T1, confined to anatomical site of origin; T2, extension and/or fixative to surrounding tissue

b Size: a, <5 cm in diameter; b, >5 cm in diameter

c Nodes: N0, regional nodes not involved; N1, regional nodes involved; Nx, regional nodes status unknown

d Metastases: M0, no distant metastases; M1, metastases present (includes positive cytology in CSF, pleural or peritoneal fluid)

**Table:** 

COG-STS risk classification									
Embryonal	I	II			III				IV
group		A	B	C	Orbit		Non-orbit		
stage		N0, Nx	N1	N1	N0, Nx	N1	N0, Nx	N1	
1 (Favorable site)	Low subset 1					Low subset 2			
2 (Unfavorable site)									
3 (Unfavorable site)	Low subset 2				Intermediate				
4 (Distant metastases)									High
Alveolar	I	II			III				IV
group		A	B	C	Orbit		Non-orbit		
stage		N0, Nx	N1	N1	N0, Nx	N1	N0, Nx	N1	
1 (Favorable site)									
2 (Unfavorable site)	Intermediate								
3 (Unfavorable site)									
4 (Distant metastases)									High

In Europe, various trials have attempted to reduce late functional and aesthetic deficits associated with local treatment, including omission of local treatment with strong induction chemotherapy, adjustment of treatment intensity based on the response to chemotherapy and adoption of delayed primary excision (DPE) to avoid initial surgical resection. Cooperation among clinical research groups was intensified in 2000, ultimately leading to the foundation of the EpSSG. Since then, the treatment based on the RMS-2005 protocol has been implemented, and many results have been published in recent years, some of which are described in the later chapter (44–46). Unlike in the USA, ifosfamide is used instead of cyclophosphamide as an alkylating agent in Europe.

Histological type is extremely important when discussing the treatment of RMS. There are major differences in the clinical characteristics, prognosis, risk classification, and irradiation dose between the embryonal and alveolar type. It is known that the alveolar type is more common in patients with head and neck primary over 10 years of age. On the other hand, patients with orbital RMS have a higher frequency of the embryonal type, which has a favourable prognosis. In the alveolar type, the *PAX3-FOXO1* or *PAX7-FOXO1* fusion genes are associated with tumorigenesis as driver mutations (25), while in the embryonal type, activation of the IGF2 pathway is related to tumorigenesis (47,48). In recent years, it has been noted that gene signatures could improve current risk stratification (49). Patients who were *PAX3-FOXO1* fusion positive had a significantly poorer outcome compared with both alveolar-negative and *PAX7-FOXO1* fusion-positive patients (50).

Head and neck RMS are anatomically classified into orbital, parameningeal and non-parameningeal lesions. Parameningeal RMS (PM-RMS) refer to tumours arising from the nasopharynx, nasal cavity, paranasal sinus, parapharyngeal space, pterygopalatine fossa, masticator space, mastoid cavity and middle ear, and are distinguished anatomically and prognostically from other head and neck RMS (51). Owing to the anatomical constraints and infiltrative nature of PM-RMS, it is challenging in 95% of cases to obtain an oncologically negative margin resection without compromising form and function. Therefore, radical resection is not appropriate for the initial surgical treatment, which is usually limited to biopsy alone. According to the guidelines from the International Soft Tissue Sarcoma Database Consortium consisting of collaboration between COG and EpSSG, VAC-based chemoradiation remains the preferred initial treatment modality for PM-RMS, with surgery limited to initial biopsy to establish the diagnosis (52). Radiotherapy is generally performed after four cycles or less of VAC chemotherapy, unless there is intracranial extension or cranial paralysis (39). Primary resection in smaller tumours has advantages such as avoidance of radiation-associated short- and long-term morbidities as described in a small series (53,54). If surgical resection is considered, smaller tumours (<5 cm, those without dural involvement) appear to be more amenable to resection relative to a larger tumours (53). These tumours are usually resected through a transfacial or cranio-orbito-zygomatic approach, which requires reconstruction after tumour resection is completed (55,56). Adjuvant chemotherapy is usually resumed within 2 weeks after surgery, and RT commences as soon as possible after adequate healing of the surgical wound, although delays are often necessary because of postoperative complications (52,57). With advances in endoscopic approaches and quality of imaging studies, certain tumours in the nasopharynx, sinonasal cavities and skull base without dural involvement may be amenable to endoscopic approaches (54,58).

The excellent prognosis for patients with orbital RMS has been demonstrated in successive studies from the IRSG and several European cooperative groups (34–36,59). Most of the patients have small tumours that are readily apparent and could be detected early. It is very rare to detect distant metastases at the time of diagnosis. The most predominant histologic subtype is embryonal type, which has a favourable prognosis relative to alveolar type (60). Because most patients have a favourable outlook for survival, many physicians limit the initial surgical procedure to a biopsy only (Group III) with emphasis on cosmetic aspects, unless the mass is easily accessible and can be grossly removed (Group II). Due to a low rate of recurrence, there are only a few series that report treatment and outcome for patients with relapse or refractory orbital RMS. In a retrospective study of orbital RMS that recurred after treatment with IRSG Protocols III/IV, 24 of 188 patients (12.8%) developed local (*n* = 22) or distant relapse (*n* = 2) (59). There was no protocol-suggested outline of treatment to consider following relapse; each group of physicians decided on a plan according to their best judgement about each patients. Various salvage treatments were performed, including surgery (at least biopsy to confirm the nature of the recurrence), chemotherapy and RT (external radiation and brachytherapy if possible). Surgical treatment included orbital extraction in 10 patients, enucleation of the globe in 2 patients, tumour excision in 3 patients and only biopsy in 1 patient. Moreover, 22 of the 24 patients (91.7%) were alive and free of recurrent tumour at a median of 6.9 years after relapse. Two patients died, both with embryonal types. Thus, even patients with local recurrence after initial treatment can be expected to be cured. Salvage treatment must be individualized according to each patient’s previous history and ability to tolerate further toxic agents and methods to achieve local control.

The optimal treatment of RMS includes systemic chemotherapy and local treatment with surgery and/or RT; however, the late effects among survivors are concerning. Approximately half of the survivors of childhood sarcomas have at least one major adverse outcome in their health status, illustrating the need to develop less toxic treatments that are still effective (61–63). To facilitate local control and reduce the total treatment intensity, induction chemotherapy followed by surgical resection has been attempted in some studies. This procedure is called DPE, and it was previously referred to as second-look operation. Patients who undergo DPE may receive reduced RT doses to minimize the long-term complications associated with high-dose RT. The combination of DPE and modulated RT may maintain local control yet reduce the incidence of chronic health conditions and secondary malignancies associated with the current treatment paradigm.

**Figure 2:**
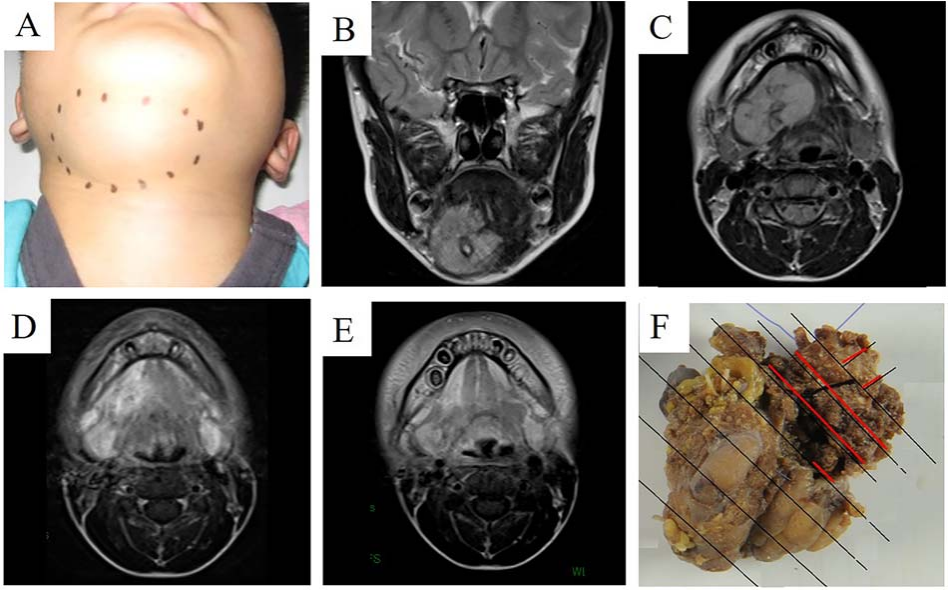
A case of alveolar rhabdomyosarcoma in a 6-year-old patient. (A–C) An invasive tumour located in the submandibular region. (D) After one cycle of VAC, the tumour dramatically shrunk. (E) After four cycles of VAC, the tumour was in complete remission on imaging. (F) Surgical specimen of a delayed primary excision (submandibular dissection). A few viable tumour cells were detected in the red lines. After DPE, VAC chemotherapy and postoperative radiotherapy were performed.

In the head and neck regions, DPE has also been investigated for non-PM-RMS (Fig. 2). In the COG D9602 trial of patients with low-risk RMS, RT dose reduction based on the completeness of surgical resection of the primary tumour after chemotherapy did not compromise local control in non-PM-RMS (38).

In the COG D9803 trial (64) for intermediate-risk RMS, patients were evaluated for response to induction chemotherapy at week 12, and surgical resection was then encouraged for the candidate anatomical site of organ preservation without loss of form or function. The postoperative RT dose was determined according to the number of residual tumours, that is, DPE 36 Gy if the tumour was completely resected with a negative margin, 41.4 Gy for microscopic residual tumour or clinical complete remission by imaging criteria with biopsy confirmation, or 50.4 Gy for those without or with DPE in which gross residual disease remained postoperatively. Unfortunately, patients with head and neck primary were not enrolled. As a result, 79% of pathology specimens obtained at DPE contained viable tumours, 84% of patients with DPE were eligible to receive a reduced RT dose as a result of gross total tumour resection and the local control rates at these selected anatomical sites were similar to IRS-IV, a study that did not include planned DPE and used higher RT doses. Currently, we lack metrics sufficient to detect clinically relevant differences in late effects in patients receiving doses of 36 vs. 50.4 Gy. However, it is reasonable to accept any reduction in RT dose, so long as adequate local control is maintained, is beneficial and desirable (64).

The EpSSG RMS 2005 study of non-PM-RMS showed the possibility of omitting RT after DPE in the low-risk group (44). Of the 21 patients who achieved complete pathological resection by DPE and did not receive postoperative RT, local recurrence was noted in only three cases. However, in the RMS2005 protocol, when postoperative RT was omitted, the dose of the alkylating agent was enhanced by nine cycles of ifosfamide, vincristine and d-actinomycin. The risk of long-term effects after major surgery and RT to the head and neck region is high (65). The best local treatment in these relatively young patients must be decided during a multidisciplinary discussion (66). The trade-off between reduced radiation dose and improved quality of life with the addition of DPE must be evaluated in future clinical trials (67).

Although improvements in treatment strategies for patients with localized RMS have occurred over the past three decades, little progress has been made in curing patients with metastatic disease (33–36,39). The presence of metastatic disease is a strong predictor of the clinical outcomes in patients with RMS. Despite aggressive multimodality treatments, these children fare poorly; only 25% are expected to be disease-free 3 years after diagnosis (34,35). In the COG ARST0431 study, the combination of VAC with irinotecan, doxorubicin, ifosfamide and etoposide improved the 3-year survival rate to 56% (68). Recurrent or refractory RMS is treated by chemotherapy with ifosfamide, carboplatin and etoposide; however, the 1-year survival rate is approximately 50% (69). As another standard second line, intravenous vinorelbine and continuous low-dose oral cyclophosphamide has been shown to be effective, with the best overall response rate of 36% (70,71). Irinotecan with vincristine (VI) are also options for recurrent RMS, with a reported 1-year recurrence-free survival rate of 38% and 1-year OS of 60% (72). Recently, the efficacy and safety of the VI–irinotecan combination with temozolomide (VIT) was also demonstrated (73). The VIT achieved significantly better OS compared with VI (adjusted hazard ratio, 0.55; 95% CI, 0.35–0.84). VIT is considered the new standard treatment in relapse or refractory RMS in Europe and will be the control arm in the next randomized trial.

(2) Osteosarcomas

Osteosarcomas accounts for approximately ≤1% of all head and neck cancers. The vast majority occur in the mandible and maxilla (74). In head and neck osteosarcomas, obtaining sufficient evidence is challenging because of its rarity and the difficulty of conducting randomized trials. It is often treated based on the evidence of osteosarcomas arising in the long bones, which is more common. Osteosarcomas are histologically heterogeneous and grow in the bone microenvironment, a very specialized, complex and highly dynamic environment composed of bone cells (osteoclasts, osteoblasts and osteocytes), stromal cells (mesenchymal stromal cells and fibroblasts), vascular cells (endothelial cells and pericytes), immune cells (macrophages and lymphocytes) and a mineralized extracellular matrix (75). The treatment strategy depends on the pathological grade.

Low-grade osteosarcomas such as parosteal osteosarcomas (76), periosteal osteosarcomas (77,78) and low-grade central osteosarcomas (79) are poorly sensitive to chemotherapy and treated by surgery. No evidence has confirmed the benefit of adjuvant therapy, such as chemotherapy or RT. Incomplete resection was associated with an increased risk of local recurrence with dedifferentiation. Dedifferentiation markedly increased the risk of metastasis and is noted in 25–35% at recurrence (76,80,81). It is critical to ensure local control during the initial surgery (76).

**Figure 3:**
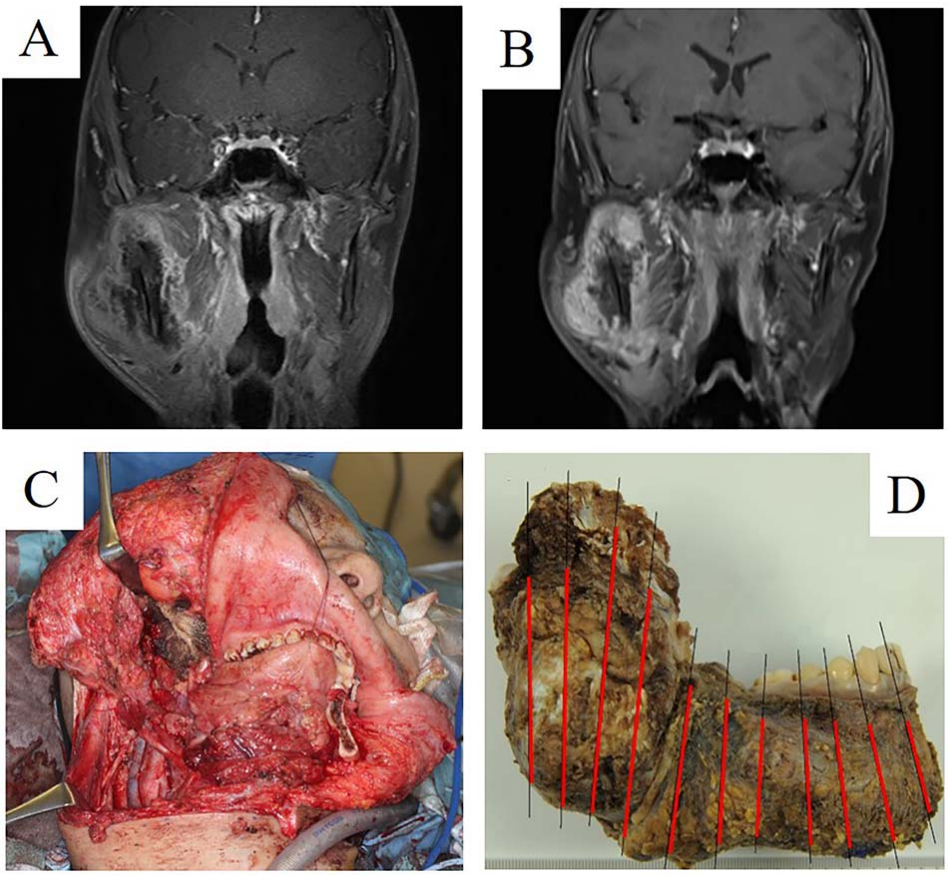
A 20-year-old woman with high-grade osteosarcoma of the mandible. (A) The tumour invaded to the masseter and pterygoid muscles. (B) Imaging results: after two cycles of MAP neoadjuvant chemotherapy. (C) Intraoperative photograph of segmental mandibulectomy. (D) Surgical specimen of segmental mandibulectomy. Viable tumour cells were detected in 70% of the area (red lines). After surgery, MAP chemotherapy and postoperative radiotherapy were performed.

High-grade osteosarcomas such as conventional osteosarcomas, telangiectatic osteosarcomas and small cell osteosarcomas are prone to distant metastasis; therefore, a multidisciplinary treatment combining surgery and perioperative chemotherapy is essential (82) (Fig. 3). The goal of preoperative chemotherapy is the treatment of micrometastases, reduction of the primary tumour and determination of the histological response to chemotherapy. Regardless of the efficacy of chemotherapy, surgical resection is mandatory. Thus, osteosarcomas arising in the mandible cannot avoid a segmental mandibulectomy. In resected specimens, if tumour necrosis is >90% after preoperative chemotherapy, the prognosis is favourable; however, if tumour necrosis is <90%, recurrence within 2 years is common (83,84).

The three-drug combination of methotrexate, doxorubicin and cisplatin (MAP regimen) had been the standard chemotherapy for osteosarcomas over the past three decades (82). The addition of ifosfamide to the MAP regimen was expected to improve prognosis; however, several clinical trials, including the EURMOS-1 trial, have failed to show any benefit (85–87).

Head and neck osteosarcomas present and behave differently from the more common long-bone osteosarcomas. Hence, it is controversial to treat based on evidence from different regions (88). First, it tends to occur in the third or fourth decades of life, compared with long-bone osteosarcomas in adolescents and young adults (89). Second, metastases occur less frequently, with the failure of treatment manifesting most commonly as local recurrence rather than distant failure, as seen in long-bone osteosarcomas (90). Third, in contrast to the good histological response to chemotherapy expected in 50% of the patients with extremity osteosarcomas (91), the highest and lowest proportion of good histological responses was reported at 35% (92) and 0% of patients with head and neck osteosarcomas (93). Fourth, no significant benefit on OS by neoadjuvant chemotherapy has been found in several retrospective studies (94–96). These findings suggest that the efficacy of neoadjuvant chemotherapy in head and neck osteosarcomas is inferior to that in long-bone osteosarcomas (88). The review of the neoadjuvant chemotherapy for head and neck osteosarcomas does not show a consistent benefit on survival or recurrence (88). This review analysed 18 articles from 264 articles published between 1940 and 2019 that were retrospective studies of five or more cases, and only three articles reported a positive impact of neoadjuvant chemotherapy on survival. From these papers, 222 patients were compared by those who had surgery with those who had neoadjuvant chemotherapy and surgery, and there was no statistical difference in disease-specific survival (*P* = 0.26). Furthermore, even when restricted to patients with intermediate- or high-grade histology, the use of neoadjuvant chemotherapy made no difference in survival (*P* = 0.37). However, most of the analysed data were relatively small sample-sized, retrospective studies. Given the rarity of this disease and difficulty in conducting randomized controlled trials, it is imperative to accumulate evidence through multicentre observational studies or the publication of raw data in repositories.

The role of second-line chemotherapy for recurrent or metastatic osteosarcomas is much less well defined (97). The treatment choice often includes ifosfamide or cyclophosphamide, possibly in association with etoposide and/or carboplatin (97–99). In general, despite second-line treatment, the prognosis of recurrent disease has remained poor, with a long-term post-relapse survival rate of <20% (100,101). Several *in vitro* and preclinical studies have demonstrated the expression of tyrosine kinase receptors such as platelet-derived growth factor receptors (PDGFRs), vascular endothelial growth factor receptors (VEGFRs) and rearranged during transfection (RET) in osteosarcomas, highlighting the relevance of multi-kinase inhibitors as potential therapeutic tools (102,103). Regorafenib, apatinib and cabozantinib showed response rates of 8–14, 43 and 12%, respectively (104–107). Osteosarcomas are genomically complex and characterized by widespread and recurrent somatic copy-number alterations and structural rearrangements. However, few patients have significant copy-number alteration gains in druggable, clinically actionable genes, despite exhibiting genome-wide copy-number alteration. Moreover, each patient showed a varying degree of loss of at least one canonical tumour suppressor gene. Thus, it is likely that several distinct oncogenic drivers are responsible for the aggressive nature of this disease in individual patients (108). This finding highlights the increasing possibility of ‘personalized’ approaches considering heterogeneous signalling pathways.

(3) Ewing’s sarcoma

**Figure 4:**
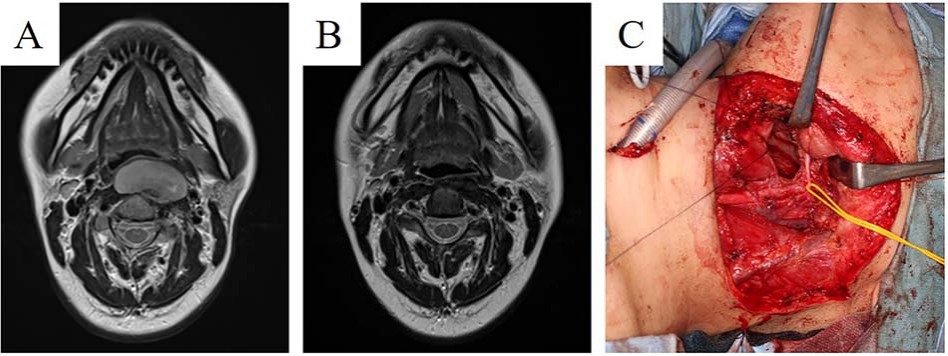
A 32-year-old woman with Ewing’s sarcoma of the pharynx. (A) A submucosal tumour invading the anterior vertebral muscles was detected on the posterior wall of the pharynx. (B) After VDC-IE neoadjuvant chemotherapy, the tumour was in complete remission on imaging. (C) Partial pharyngectomy and free flap reconstruction were performed. The resected specimen revealed no viable tumour cells. After surgery, VDC-IE chemotherapy was performed.

Because 20–30% of patients with Ewing’s sarcoma have evidence of metastasis at diagnosis, multidisciplinary treatment with local treatment by surgery, radiation and systemic treatment with multi-agent chemotherapy is the basic approach (109) (Fig. 4). Although the tumour is radiosensitive, the local or combined (local recurrence and systemic metastasis) recurrence rates after surgery with or without RT were significantly lower than those after definitive RT, suggesting that surgery is preferred for resectable cases (110). Moreover, given the high rates of local recurrence in inadequate resection margins with microscopic residual disease, even with postoperative RT, wide resection is the standard approach (111). Patients with extensive resection and good histologic responses to neoadjuvant chemotherapy did well, with 1 of 101 (1%) local and combined relapses without postoperative RT, whereas patients with poor histologic response rates failed locally in 3 of 25 cases (12%) (111). Thus, postoperative RT can be omitted for good responders to chemotherapy, who are eligible for wide excision; however, for poor responders to chemotherapy, postoperative RT improves local control, even if wide excision is possible. Generally, four to six cycles of neoadjuvant chemotherapy are administered.

Owing to disease rarity, most works on Ewing’s sarcoma in the head and neck are retrospective studies. Even in the head and neck region, complete resection should always be attempted; however, complete resection is difficult to achieve in the head and neck regions without significant cosmetic or functional defects, and radiation is nearly always required (112,113). Alkylating agent-based chemoradiation may be a reasonable alternative to surgery for treating tumours that require an unacceptable degree of resection; however, 5-year estimates of the cumulative incidences of local failure were 71 ± 13% for patients who underwent chemoradiation due to incomplete resection or biopsy alone (112–114).

Evidence for Ewing’s sarcoma has been established by clinical trials conducted in the USA and Europe. In the USA, the Intergroup Ewing Sarcoma Study-1,2 showed the benefit of adding doxorubicin to VAC (115). Subsequently, a study conducted by the National Cancer Institute (NCI), NCI INT-0091, demonstrated that vincristine, dactinomycin, cyclophosphamide and doxorubicin (VADC) alternating with ifosfamide and etoposide (IE) were superior to VADC alone (116), establishing the basis for the current standard regimen. Further regimen modifications were attempted, and in the NCI INT-0154 study, a VDC-IE therapy with increased doses of alkylating agents (cyclophosphamide and ifosfamide) was performed, but did not improve the prognosis (117). The AEW0031 trial tested whether treatment efficacy could be improved by shortening the treatment duration rather than modifying the drug dose, and using G-CSF reduced the treatment duration from 3 to 2 weeks, which was shown to improve the disease-free survival (118).

Regimens using ifosfamide as an alkylating agent have been developed in Europe, and the CFSS86 trial demonstrated the efficacy of VADC in the standard risk group, such as small extremity lesions and vincristine, dactinomycin, ifosfamide and doxorubicin (VAIA) in the high-risk group, such as lesions with tumour volume >100 mL and/or at central-axis primary lesions (119). In addition, chemoresponders had significantly higher 10-year disease-free survival rates, indicating that chemoresponse is a prognostic factor (119). The subsequent EICFSS92 study showed the benefit of adding etoposide to VAIA for the CFSS86 high-risk group (120). Based on the Euro-Ewing99 trial, until recently, vincristine, ifosfamide, doxorubicin and etoposide (VIDE) was the standard regimen (121); however, in the Euro-Ewing2012 study, VIDE and VDC/IE regimens were compared (122,123). At a median follow-up of 47 months, VDC/IE improved event-free survival and OS to VIDE (3-year event-free survival 67 vs. 61%, HR 0.71, 95% CI 0.55–0.92; 3-year OS 82 vs. 74%, HR 0.62, 95% CI 0.46–0.85). VDC/IE is more effective, less toxic and shorter in duration for all stages of newly diagnosed Ewing’s sarcoma than VIDC induction and should now be the standard of care for Ewing’s sarcoma.

In metastatic cases, highly effective chemotherapy has not been established, and even with multimodality therapy using VDC-IE and VIDE, the 5-year OS rate is approximately 20% (124,125).

(4) Angiosarcomas

Angiosarcomas can arise anywhere in the body, and it most commonly presents as a cutaneous disease in older white men, involving the head and neck, particularly the scalp (126). A retrospective study of 70 cases of non-metastatic face and scalp angiosarcomas revealed that combined modality local therapy, including surgery and RT, improved local control, disease-specific survival and OS compared with monotherapy of surgery or RT (127). Furthermore, a meta-analysis of 379 cases reported that age <70 years, tumour diameter <5 cm, primary facial tumour and surgical treatment were favourable prognostic factors of survival. Surgery and postoperative RT are considered effective treatment approaches (126). A retrospective study of resectable lesions in younger patients showed that a multimodality therapy with surgery and RT and/or chemotherapy was the sole independent prognostic factor associated with improved OS (128). Taxane-based chemoradiation, followed by maintenance chemotherapy, has demonstrated efficacy in locally advanced unresectable angiosarcomas (129).

Anthracyclines, ifosfamide and taxanes are commonly used to treat unresectable, metastatic angiosarcomas. In retrospective studies, the response rates for anthracyclines and taxanes reported were 33 and 18.5%, respectively (126,130).

## Novel drugs for recurrent or metastatic soft tissue sarcomas

Non-round cell soft tissue sarcomas are morphologically composed of spindle-shaped or pleomorphic cells that are resistant to chemotherapy. They include liposarcomas, leiomyosarcomas and synovial sarcomas. The chemotherapy for non-round cell soft tissue sarcomas, in which standard chemotherapy has not been established, includes doxorubicin, ifosfamide, dacarbazine and gemcitabine. Among these, doxorubicin and ifosfamide have good response rates even as monotherapy, and doxorubicin-based regimens had been developed in the view of toxicity. However, a meta-analysis comparing doxorubicin-based combination therapy with doxorubicin monotherapy showed that combination therapy increased adverse effects but did not improve OS; the median survival for doxorubicin alone for recurrent or metastatic sarcomas was 7.7–12 months (4). Doxorubicin monotherapy is an appropriate first-line chemotherapy option for advanced or metastatic soft tissue sarcomas.

Currently, doxorubicin monotherapy remains the first-line regimen for unresectable advanced or recurrent soft tissue sarcomas; however, evidence of second-line regimen for specific tissue types in novel agents such as pazopanib, trabectedin and eribulin has recently been established. A typical pivot trial of the three agents is shown in Table 2.

**Table 2:** Pivot trials and major retrospective studies in pazopanib, trabectedin and eribulin

Histology/study design (Ref)	Experimental (*n*)/control (*n*)	Response rate	Progression-free survival	Overall survival
Pazopanib				
LPS, LMS, SS, other STS^*^/	Pazopanib (140)	PR 6%	LPS 80 D, LMS 91 D,	LPS 197 D, LMS 354 D
EORTC62043 phase II (131)			SS 161 D, others 91 D	SS 310 D, others 299 D
LMS, SS, other STS^*^/	Pazopanib (246)/	Pazpanive: PR 6%, SD 67%	Pazopanib: 4.6 M	Pazopanib: 12.5 M
PALETTE phase III (5)	Placebo (121)	Placebo: SD 38%	Placebo: 1.6 M	Placebo: 10.7 M
Intermediate–high-grade LPS/	Pazopanib (41)	PR 2.4%	4.44 M	12.6 M
Phase II (132)		SD 41.5%		
Solitary fibrous tumour/	Pazopanib (36)	PR 51%	5.57 M	2y OS 73%
Phase II (133)		SD 26%		
Alveolar soft part sarcoma/	Pazopanib (6)	PR 16.7%	5.5 M	32 M
Phase II (134)		SD 83.3%		
Trabectedin				
LPS, LMS/	Trabectedin (345)/	Trabectedin: ORR 9.9%	Trabectedin: 4.2 M	Trabectedin: 12.4 M
ET743-SAR-3007 phase III (6)	Dacarbazin (171)	Dacarbazin: ORR 6.9%	Dacarbazin: 1.5 M	Dacarbazin: 12.9 M
LPS, LMS, SS, US, MFS/	Trabectedin (51)/	Trabectedin: ORR 13.7%	Trabectedin: 3.1 M	Trabectedin: 13.6 M
T-SAR phase III (138)	BSC (50)	BSC: ORR 0%	BSC: 1.5 M	BSC: 10.8 M
Myxoid liposarcoma/	Trabectedin (50)/	CR 3.9%	14 M	
Retrospective study (139)		PR 47%	6 M PFS 88%	
Translocation-related sarcoma/	Trabectedin (39)/	Trabectedin: PR 11%, SD 8%, PD 19%	Trabectedin: 5.6 M	
JapicCTI-121850 phase II (140)	BSC (37)	BSC: PR 0%, SD 0%, PD 47%	BSC: 0.9 M	
Translocation-related sarcoma/	Trabectedin (60)/	Trabectedin: ORR^*^^*^ 5.9%	Trabectedin: 16.1 M	HR = 0.77 (95% CI, 0.4–1.4)
NCT00796120 phase III (141)	Doxorubicin (59)	Doxorubicin: ORR^*^^*^ 27.0%	Doxorubicin: 8.8 M	
Eribulin				
LPS, LMS/	Eribulin (228)/	Eribulin: ORR 4%	Eribulin: 2.6 M	Eribulin: 13.5 M
E3789-G000-309 phase III (143)	Dacarbazin (224)	Dacarbazin: ORR 5%	Dacarbazin: 2.6 M	Dacarbazin: 11.5 M
LPS/	Eribulin (70)/	Eribulin: PR 1.4%, SD 64.8%	Eribulin: 2.9 M	Eribulin: 15.6 M
E3789-G000-309 phase III (144)	Dacarbazin (71)	Dacarbazin: PR 0%, SD 44.4%	Dacarbazin: 1.7 M	Dacarbazin: 8.4 M

LPS, liposarcoma; LMS, leiomyosarcoma; SS, synovial sarcoma; US, undifferentiated sarcoma; MFS, myxofibrosarcoma; PR, partial response; SD, stable disease

ORR, objective response rate; BSC, best supportive care; ORR^*^^*^, objective response rate in efficacy population; Ref, reference; *n*, number of patients; M, months; D, days; PFS, progression-free survival; OS, overall survival

^*^Patients with the following sarcoma entities were excluded: embryonal rhabdomyosarcoma, chondrosarcoma, osteosarcoma, Gastrointestinal Stromal Tumor (GISTs), dermatofibrosarcoma protuberans, Ewing sarcoma/primitive neuroectodermal tumours, inflammatory myofibroblastic tumour and malignant mesothelioma

Pazopanib is an oral angiogenesis inhibitor that targets, among other receptors, VEGFRs, PDGFRs and c-kit. The EORTC 62043 study (131) and the PALETTE study (5) showed prolonged OS with pazopanib as second-line chemotherapy in patients with metastatic non-adipocytic soft tissue sarcomas, such as leiomyosarcomas and synovial sarcomas, after failed first-line chemotherapy. Subsequent phase II trials have also reported efficacy in liposarcoma subsets, particularly high-grade liposarcomas, which were excluded in the PALETTE trial (132). Recently, some clinical trials have suggested that pazopanib could be an option for the systemic treatment of solitary fibrous tumours without dedifferentiation (133) or alveolar soft tissue sarcomas (134,135).

Trabectedin, an alkylating agent, causes DNA structural changes by selectively binding to the minor groove of the DNA. Consequently, this inhibits the DNA repair mechanism and induces apoptosis (136). In addition, as a specific mechanism against some translocation-related sarcomas, it suppresses the function of the fusion protein as a transcription factor and regulates the expression of cancer-related genes (137). A phase III trial, which compared trabectedin with dacarbazine in patients with advanced liposarcomas or leiomyosarcomas after prior treatment with an anthracycline, showed the superiority of trabectedin in progression-free survival (PFS) (6). Trabectedin is expected to be effective as a second-line chemotherapeutic agent for liposarcomas and leiomyosarcomas (138). A retrospective study in Europe showed a favourable response rate of 51% and a median PFS of 14 months in myxoid liposarcomas, a subtype of liposarcomas with chromosomal translocation *t*(12;16) (q13;p11) or *t*(12;22) (q13;p12) (139). Furthermore, a multicentre, open-label phase II study in Japan for translocation-related sarcomas also reported favourable outcomes (140). In a randomized phase III trial comparing trabectedin and doxorubicin as a first line in advanced/metastatic translocation-related sarcomas, there was no statistically significant difference in PFS between the two groups. However, considering the limitation of the study, which included high rate of censoring, trabectedin showed efficacy and safety in this population and setting (141).

Eribulin is a tubulin inhibitor similar to taxane. Taxane binds to the interior surface of microtubules, whereas eribulin binds to the plus end of the microtubules (142). In a global phase III/309 trial conducted in patients with liposarcomas and leiomyosarcomas who had at least two prior chemotherapy regimens, including an anthracycline, eribulin showed OS advantage compared with dacarbazine (143), and a subgroup analysis showed a significant benefit in liposarcomas, especially de-differentiated liposarcomas (144).

## Proton and carbon ion radiotherapy for head and neck sarcomas

In recent years, charged particles (e.g. protons and carbon ions) have been used in RT for sarcomas. Photons (X-rays) emit their maximal energy near the body’s surface, which gradually decreases as they travel deeper into the body. In contrast, charged particles deposit a relatively low dose near the body’s surface and emit their maximum energy just before they stop inside the body (145,146). This effect, known as the Bragg peak effect, facilitates delivery of an optimal dose to the tumour while exposing critical organs surrounding the tumour to lower doses. The biological effectiveness of proton and carbon ion beams differs. Proton beams deliver an equivalent mean energy per unit length of their trajectory to body tissues as photon beams, whereas carbon ion beams deliver a three-fold larger mean energy per unit length of their trajectory to body tissues compared to photon and proton beams (147). Given these physical properties, the primary advantage of proton RT is reducing acute and late toxicity by decreasing the amount of normal tissue irradiated in neoadjuvant or adjuvant settings (148). However, because carbon ion beams have a higher biological effectiveness than proton beams, they are used as definitive RT for locally advanced, radioresistant sarcomas (149–151). The available clinical research data for these methods are shown in Table 3.

**Table 3:** Major clinical studies of proton and carbon ion radiotherapy

Radiotherapy	Histology	Primary (*n*)	Resectability	Study design	Local control	Overall survival	Toxicity (*n*)	Ref
Proton	Rhabdomyosarcoma	PM (27), orbit (13),	Resectable/unresectable	Phase II	5y 81%	5y 78%	Acute G3 (13), G4 (0)	154
		HN (4), others (13)					Late G3 (3), G4 (0)	
Proton	Ewing’s sarcoma	Skull base (3), HN (3)	Resectable/unresectable	Retrospective	3y 86%	3y 89%	Acute G3 (7), G4 (0)	155
		Orbit (2), others (22)					Late G3/G4 (5)	
Proton	Chordoma/	Skull base (67)/	Resectable/unresectable	Retrospective	5y 71%	5y 75%	Acute G3 (9)	158
	Chondrosarcoma	CS (8), others (20)					Late G3/G4 (9)	
Carbon ion	Osteosarcoma	Trunk (77)	Unresectable	Retrospective	5y 62%	3y 33%	Acute and late G3/G4 (3)	161
								
Carbon ion	Osteosarcoma	Trunk (26)	Unresectable	Retrospective	5y 63%	3y 42%	Acute (0)	162
							Late G3/4 (4)	
Proton vs. carbon	Chondrosarcoma	Skull base (100)	Unresectable	Retrospective	Proton: 4y 100%	Proton: 4y 100%	Proton: acute/late G3/4 (0)	163
					Carbon: 4y 91%	Carbon: 4y 93%	Carbon: acute/late G3/4 (0)	
Proton vs. carbon	Chondrosarcoma	Skull base (133)	Resectable/unresectable	Prospective	Proton: 5y 84%	Proton: 5y 83%	Proton: 3y HGT-FS 91%	168
					Carbon: 5y 71%	Carbon: 5y 82%	Carbon: 3y HGT-FS 85%	

*n*, Number of patients; Ref, reference; PM, parameningeal; HN, head and neck; y, years; G, grade; CS, cervical spine; HGT-FS, high-grade toxicity-free survival

Sarcomas in children, such as RMS and Ewing’s sarcoma, are generally radiosensitive and show improved outcomes with multidisciplinary therapy. Therefore, the main goal of proton RT is to reduce the incidence and severity of late post-treatment effects rather than to improve treatment outcomes (152). Indeed, clinical data from mixed paediatric and adult samples of more than 1000 patients suggest a reduction in second tumour rates in the proton-treated group compared with the photon-treated group (153). Table 3 presents clinical data on the multidisciplinary treatment of radiosensitive sarcomas using proton RT. A phase II trial of 57 paediatric RMS patients—including parameningeal primary cases—reported 5-year OS and local control rates of 78% and 81%, respectively, for the entire cohort. Early findings from this phase II trial indicate that disease outcomes in photon-treated populations are similar to historical controls, with only minor treatment-related adverse effects (152). A retrospective study of 30 children with Ewing’s sarcoma also showed that proton RT is well tolerated, with few reports of acute toxicity and low late toxicity rates (153). Relatively radioresistant bone sarcomas of the skull base and spine, especially chordomas and chondrosarcomas that are difficult to surgically resect because of their anatomical features, are often the target of proton RT (154). Because chordomas and chondrosarcomas respond best to doses higher than 70 Gy (155), proton RT is effective in the skull base region proximal to dose-limited neural structures (brainstem, spinal cord, optic nerve structures, etc.) (154,156). Table 3 presents the clinical data on the multidisciplinary treatment of radioresistant sarcomas with proton RT. In a retrospective analysis of 96 non-metastatic patients with chordoma or chondrosarcoma, the 5-year OS and local control rates were 75 and 71%, respectively. Acute grade 3 and late ≥grade 3 toxicities were observed in nine patients (9.4%) each (156).

As one of the more advanced radiation modalities, carbon ion RT is a promising treatment strategy for sarcomas because carbon ion beams have a greater killing effect on tumour cells than proton beams (151,157). Most bone sarcoma types (e.g. osteosarcomas, chondrosarcomas and chordomas) are resistant to radiation, thereby requiring higher doses of RT to achieve adequate local control (158). Table 3 shows a retrospective clinical study of carbon ion RT for unresectable radioresistant sarcoma. For osteosarcoma, the 5-year survival and 5-year local control rates were 33–50% and 62–63%, respectively (159,160), whereas for chondrosarcoma, they were 92% and 90%, respectively (161).

In general, late high-grade (grades 3–4) toxicity rates are reported to be 6–8.1% and 4.1–6% for protons and carbon ions, respectively (162–165). Temporal lobe necrosis is one of the most dreaded late adverse events in high-dose charged particle RT for skull base tumours. It is reported that 10.4–28% of clinical and radiological MRI changes are consistent with radiation necrosis after proton or carbon ion RT (166,167). Carbon ion beams have a greater biological effect than proton beams, which may increase treatment efficacy (168). However, there is concern about damage to the surrounding normal tissues due to the strong biological effects of carbon ion beams. The choice between proton and carbon ion beams for skull base sarcomas is controversial (168,169) because the strength of the biological effect alone does not determine local control or outcome. In a retrospective study comparing the results of RT with protons and carbon ions in 101 patients with skull base chondrosarcoma, the 4-year local control rate was 100% for protons and 90.5% for carbon ions, whereas the 4-year OS rate was 100% for protons and 92.9% for carbon ions, with no toxicity worse than grade 3 (163). No significant difference was found between carbon ions and protons in these initial results. In the largest prospective study to date of dual particle radiation treatment for 135 patients with skull base chondrosarcoma, the 5-year local control rate was 84% for protons and 71% for carbon ions (166). Carbon ion RT is the treatment of choice in cases of unfavourable tumour characteristics, which may negatively influence the local control rate. Previous studies have reported no significant differences in treatment outcomes and adverse events between proton RT and carbon ion RT over the short term (Table 3). However, the level of evidence is limited because of the study design, several biases and possible confounders (i.e. differences in patient numbers and follow-up periods). Further evidence is currently being gathered through a randomized phase III non-inferiority trial comparing protons with carbon ions in patients with bone sarcoma of the skull base; this trial is still recruiting (170,171).

## Conclusion

Head and neck sarcomas are rare tumours. Various histological types are influenced by the patient’s age and anatomical location. Histological, immunohistological and molecular analyses are important for pathological diagnosis. Needle biopsy is also expected to provide a diagnostic tool. In the multidisciplinary treatment of sarcomas, surgical resection followed by RT is the basic local treatment for resectable ones. In sarcomas that are sensitive to chemotherapy, perioperative chemotherapy is considered, and the treatment strategy is based on the recurrence risk. In advanced or metastatic soft tissue sarcomas without established standard chemotherapy, docetaxel monotherapy is the standard regimen; however, recently, the efficacy of novel agents such as pazopanib, trabectin and eribulin has been established for specific histological types. Moreover, the effectiveness of advanced radiation therapy using proton and carbon ion beams is also being established. Sarcomas in the head and neck region have different anatomical and biological characteristics from those in other regions; thus, further evidence is warranted for this rare tumour.
